# Supplementary material for: The Impact of COVID-19 on the Lifestyles of University Students: A Spanish Online Survey
Source: Healthcare (Basel). 2022 Feb 5;10(2):309. doi: 10.3390/healthcare10020309 (PMC8871949; doi:10.3390/healthcare10020309)
Supplement: Supplementary file 1 [file healthcare-10-00309-s001.zip › healthcare-1581851-supplementary.pdf]

Table S1. STROBE Statement—Checklist of items that should be included in reports of cohort studies

	Item No	Recommendation	Page: rows
**Title and abstract**	1	(*a*) Indicate the study's design with a commonly used term in the title or the abstract	Page 1: row 2
		(*b*) Provide in the abstract an informative and balanced summary of what was done and what was found	Page 1: rows 12-28
		**Introduction**	
Background/rationale	2	Explain the scientific background and rationale for the investigation being reported	Page 1-2: rows 32-95
Objectives	3	State specific objectives, including any prespecified hypotheses	Page 2: rows 95-97<br>*There are no hypotheses*
		**Methods**	
Study design	4	Present key elements of study design early in the paper	Page 3: rows 99-103
Setting	5	Describe the setting, locations, and relevant dates, including periods of recruitment, exposure, follow-up, and data collection	Page 3: rows 104-110<br>*There are no follow-up*
Participants	6	(*a*) Give the eligibility criteria, and the sources and methods of selection of participants.	Pages 3: rows 122-129
Variables	7	Clearly define all outcomes, exposures, predictors, potential confounders, and effect modifiers. Give diagnostic criteria, if applicable	Page 4: rows 146-159
Data sources/ measurement	8*	For each variable of interest, give sources of data and details of methods of assessment (measurement). Describe comparability of assessment methods if there is more than one group	Page 4: rows 159-173
Bias	9	Describe any efforts to address potential sources of bias	Not applicable
Study size	10	Explain how the study size was arrived at	Page 3: rows 114-121
Quantitative variables	11	Explain how quantitative variables were handled in the analyses.<br><br>If applicable, describe which groupings were chosen and why	Page 4: rows 175-181
Statistical methods	12	(*a*) Describe all statistical methods, including those used to control for confounding	Page 4: rows 181-191
		(*b*) Describe any methods used to examine subgroups and interactions	Page 4: rows 185-190
		(*c*) Explain how missing data were addressed	Page 4: row 192
		(*d*) If applicable, explain how loss to follow-up was addressed	Not applicable
		(*e*) Describe any sensitivity analyses	There are no sensitivity analyses
		**Results**	
Participants	13*	(a) Report numbers of individuals at each stage of study—eg numbers potentially eligible, examined for eligibility, confirmed eligible, included in the study, completing follow-up, and analysed	Page 5: rows 208-209
		(b) Give reasons for non-participation at each stage	Page 5: rows 208-209
		(c) Consider use of a flow diagram	It is not necessary
Descriptive data	14*	(a) Give characteristics of study participants (eg demographic, clinical, social) and information on exposures and potential confounders	Page 5: rows 209-221<br>Table 1

		(b) Indicate number of participants with missing data for each variable of interest	Table 1
Outcome data	15*	Report numbers of outcome events or summary measures	Page 6 to 9: rows 224-283
Main results	16	(*a*) Give unadjusted estimates and, if applicable, confounder-adjusted estimates and their precision (eg, 95% confidence interval). Make clear which confounders were adjusted for and why they were included	Tables 2, 3 and 4
		(*b*) Report category boundaries when continuous variables were categorized	Table 1
		(*c*) If relevant, consider translating estimates of relative risk into absolute risk for a meaningful time period	Not applicable
Other analyses	17	Report other analyses done—eg analyses of subgroups and interactions, and sensitivity analyses	Not applicable
		**Discussion**	
Key results	18	Summarise key results with reference to study objectives	Pages 9: rows 286-295
Limitations	19	Discuss limitations of the study, taking into account sources of potential bias or imprecision. Discuss both direction and magnitude of any potential bias	Page 11: rows 395-399
Interpretation	20	Give a cautious overall interpretation of results considering objectives, limitations, multiplicity of analyses, results from similar studies, and other relevant evidence	Pages 9-11: rows 296-381
Generalisability	21	Discuss the generalisability (external validity) of the study results	Pages 11: rows 383-394
		**Other information**	
Funding	22	Give the source of funding and the role of the funders for the present study and, if applicable, for the original study on which the present article is based	The study is not funded.
